# Revisiting the Nursing Academic Workforce Shortage: Where to From Here?

**DOI:** 10.1111/jan.16499

**Published:** 2024-10-02

**Authors:** Carmel Bond, Debra Jackson

**Affiliations:** ^1^ Sheffield Hallam University Sheffield UK; ^2^ University of Sydney Sydney New South Wales Australia

## 1 Shortage of Academic Nurses

For a number of years there has been an international shortage of academic nurses. This shortage has been exacerbated by various factors, including the ageing academic workforce, with many nurse academics approaching retirement age, meaning there will continue to be an exodus of skills and experience from the nursing academic environment. This issue, observed globally, will lead to a loss of skills and experience, such as in the United States of America (USA), where one‐third of nursing faculty is expected to retire by 2025 (AACN 2024).

This demographic trend is compounding an already serious shortage of nursing faculty, limiting the ability to enrol and educate enough new students to meet the growing demand for nursing graduates, and intensified by the fact that nursing schools in many parts of the world are under significant pressure. For instance, in the United Kingdom (UK), recruitment freezes, staffing restructures, and redundancies have caused the academic nursing workforce to shrink, heightening pressures on the remaining faculty staff (The Royal College of Nursing [RCN] 2024a). In this commentary, we will consider the continuing shortage of academic nurses and the need for strategic institutional and policy interventions to attract and retain qualified nurse academics.

## 2 Challenges in Attracting and Nurturing Talent

The future of nursing depends on the quality of educational support and preparation provided for nurses, which necessitates a strong, robust, and highly skilled academic nursing workforce. In a recent editorial in the *Journal of Advanced Nursing*, Davidson (2024) emphasised that world‐class nursing schools are built by attracting and retaining high‐calibre academic and professional staff, as these individuals shape the institution's culture. Exceptional nursing schools focus on recruiting and nurturing talent whilst promoting thought leadership in the discipline. Davidson also highlighted the importance of having research‐savvy, doctorally qualified faculty to advance the nursing profession and recognising the vital contributions of professional staff to the institution's overall success.

Attracting a strong field of applicants for academic nursing positions presents a significant challenge, extending to senior academic roles (McKenna and Thompson 2024). Several factors may account for this, for example, the wage disparity between clinical nursing roles and academic positions. In the UK, nurses in specialised or senior positions can earn higher salaries through NHS bands (a senior‐level nurse can earn from £ 105,385), overtime, and unsocial hours bonuses (The Royal College of Nursing [RCN] 2024b). In contrast, academic positions lack such additional financial incentives, which may serve as a deterrent or contribute to the reduced appeal of academic careers. Additionally, some nurses may perceive academic roles as less satisfying or impactful compared to clinical work, where patient interaction and care are central. Many nursing schools also suffer from insufficient resources and a less‐than‐optimal staff mix to adequately meet the demands of teaching, governance, and research. The potential for burnout and stress due to the high demands of academic roles (Singh et al. 2022; Zangaro et al. 2023), combined with lower levels of job satisfaction compared to clinical work, may further discourage nurses from pursuing careers in academia (Boamah et al. 2023).

Academic success is crucial for enhancing external perceptions of quality in academia. The academic environment emphasises measurable successes across various domains; however, this focus on success can lead to social comparison and workplace envy, driven by competition over positions, salaries, and other personal factors such as narcissism, self‐efficacy, and self‐esteem which have been identified as antecedents of workplace envy that can undermine professional relationships (Romero 2022) and foster a toxic work environment, resulting in incivility, aggressive behaviours, and negative outcomes. Incivility in the workplace is pervasive and nursing academia is no exception (Park and Kang 2023).

Previously, there have been calls to action highlighting the crucial need to prioritise succession planning for future nursing faculty and to enhance clinical career pathways for nurses. The current shortage of suitably qualified nurse academics and leaders underscores the need for strategic planning to develop a robust pipeline of future nurse academics and researchers (Ryder et al. 2022). Ryder et al. (2022) highlight the necessity of creating a steady stream of well‐prepared, diverse nurse scientists from early to senior career stages.

## 3 Nursing Academia: Education, Mentorship, and Research

There must be a continued focus on providing high‐quality education in nursing as education is the cornerstone of excellence in nursing practice and research, ensuring that nurses are equipped with the knowledge and skills to provide the highest levels of care and to strengthen the knowledge base upon which nursing sits. A robust educational foundation fosters desired and essential skills such as critical thinking, political astuteness, patient advocacy, evidence‐based practice, and innovation, all essential for advancing patient care outcomes and healthcare systems. Well‐educated nurses lead and contribute to groundbreaking research, driving the development of new knowledge that can inform new approaches to healthcare delivery. As the healthcare landscape evolves, the need for skilled and highly educated nurses is even more critical, positioning nursing to meet future challenges. Therefore, investing in high‐quality nursing education secures the future of nursing, strengthening the role of nurses and nursing in shaping global health.

The transition from clinician to academic is known to be a challenging experience with new academic nurses needing support as they transition into their new roles (Barken and Robstad 2024). Thus, experienced doctorally qualified academic nurses need to serve as critical friends and mentors to new academic nurses. In addition, these experienced academics often carry the lion's share of governance roles, higher‐level activities such as doctoral training and supervision, mentoring and support for activities such as publications, whilst also trying to develop their own research—in addition to teaching and other activities. These types of pressures can negatively affect the quality of the nursing academic workplace, particularly where there are inadequate numbers of suitably qualified personnel to provide crucial mentoring and support to colleagues.

Currently, there is a continuing global shortage of PhD‐prepared nurses, meaning that many educational institutions continue to employ nurses without doctoral degrees as academics (Watson, Hayter, and Jackson 2021). Some academics prioritise obtaining a teaching qualification and pursue a ‘teaching only’ career trajectory rather than a mixed teaching and research pathway. Even individuals on a ‘balanced’ teaching and research track frequently participate in minimal or no research activities, and they often have limited involvement in advanced research training or progress towards becoming active researchers. It must be noted that many academics do work in resource constrained environments, meaning that there may be little tangible support for research activity, despite it being an expected part of the academic role. However, McKenna and Thompson (2024) note a significant increase in the number of ‘teaching only’ academics in recent years in the United Kingdom, and this is also the case in other parts of the world, including Australia. This trend further reduces the capacity for nurse academics to be active as researchers.

## 4 Advancing Nursing Research

It is crucial that nursing continues to develop as a research‐based discipline. Research drives improved clinical practice, which enhances patient outcomes by ensuring that nursing care is underpinned by appropriate evidence. It also fosters innovation in healthcare, enabling nurses to address emerging challenges and adapt to the evolving demands of the profession. Additionally, a strong research foundation elevates the professional credibility of nursing, positioning nursing as a critical contributor to interdisciplinary healthcare teams. Research empowers nurse academics to continually refine and enhance curricula, ensuring that future nurses are well‐prepared to deliver high‐quality care in complex environments. Furthermore, nursing research findings contribute to shaping healthcare policy, as well as to strengthening clinical practice.

McKenna and Thompson (2024) have recently argued that while nursing research has developed in sophistication and quality over the past decades, they feel ‘a growing sense of unease that nursing research in some countries may have reached a plateau, and its future is threatened’. They have these concerns for a range of reasons, including increasing numbers of students meaning nurse academics have less time to pursue research activities, changes to curricula meaning that students have less exposure to research and fewer opportunities to undertake research, lack of funding and other infrastructure support for nurse researchers, models of doctoral training that have limited research activity, and increasing numbers of teaching only personnel in schools of nursing. Writing from an Australian perspective, Stirling et al. (2024) also highlight the issue of low levels of funding to support nursing research.

## 5 Where to From Here?

So, where can we go from here? Addressing academic workforce challenges is crucial for the continued development of nursing. The current state of the nursing academic workforce presents significant challenges that need to be urgently addressed to ensure the future of nursing education and healthcare. The faculty shortage, ageing workforces, wage disparities and heavy workloads all contribute to the difficulties in maintaining a robust academic workforce. Addressing the shortage of doctorally prepared nurses is essential as this poses a real threat to the academic pipeline, jeopardising the ability of nursing to educate and prepare future nursing students, and affecting the standing of nursing within academic institutions.

Strategic institutional and policy interventions are needed to attract and retain qualified nurse academics capable of meeting the demands of teaching, research, governance, and leadership. Supporting early‐career nurses through doctoral education will build research capacity, foster knowledge exchange, and promote inter‐professional collaboration. Nursing schools must be adequately resourced to ensure a balanced skill mix for quality teaching and meaningful research. While these efforts are crucial for sustaining nursing programs and advancing the profession, current pressures on universities may hinder their implementation, negatively affecting the student experience and the future of nursing.

## Conflicts of Interest

The authors declare no conflicts of interest.

## Data Availability

Data sharing is not applicable to this article as no new data were created or analyzed in this study.
